# Teaching Prescribing: Just What the Doctor Ordered? A Thematic Analysis of the Views of Newly Qualified Doctors

**DOI:** 10.3390/pharmacy5020032

**Published:** 2017-06-13

**Authors:** Christina R. Hansen, Elaine K. Walsh, Colin P. Bradley, Laura J. Sahm

**Affiliations:** 1 Pharmaceutical Care Research Group, School of Pharmacy, University College Cork, Cork T12 YN60, Ireland; l.sahm@ucc.ie; 2 Department of General Practice, University College Cork, Cork T12 XF62, Ireland; elaine.walsh@ucc.ie (E.K.W.); c.bradley@ucc.ie (C.P.B.); 3 Pharmacy Department, Mercy University Hospital, Cork T12 WE28, Ireland

**Keywords:** intern doctors, prescribing, education, medical curriculum, pharmacist, interdisciplinary education

## Abstract

Undergraduate medical education has been criticised for failing to adequately prepare doctors for the task of prescribing. Pharmacists have been shown to improve medication use in hospitals. This study aims to elicit the views of intern doctors on the challenges of prescribing, and to suggest changes in education to enhance prescribing practice and potential role of the pharmacist. Semi-structured, qualitative interviews were conducted with intern doctors in their first year post qualification in an Irish hospital. Data collection was conducted until no new themes emerged and thematic analysis was performed. Thirteen interviews took place. Interns described training in practical prescribing as limited and felt the curriculum failed to convey the reality of actual prescribing. Pharmacists were perceived to be a useful, but underutilised, information source in the prescribing process. They requested an earlier introduction, and repeated exposure, to prescribing, and suggested the involvement of peers and pharmacists in this teaching. Intern doctors reported difficulties in applying knowledge gained in medical school to clinical practice. New strategies are needed to enhance the clinical relevance of the medical curriculum by rethinking the learning outcomes regarding prescribing practice and the involvement of pharmacists in prescribing education.

## 1. Introduction

Influenced by changing demographics resulting in increasing numbers of older patients and more complex treatment regimens, prescribing has become an even more demanding task requiring a wide range of clinical skills and pharmacological knowledge. There are numerous associated challenges including busy clinical environments and deficits in education [1]. Prescribing is generally a task that lies within the remit of the medical profession and is a responsibility undertaken by intern doctors from their first day of internship [2]. Intern doctors have limited experience but are often responsible for the majority of prescribing in hospital, especially at the point of discharge. If one compares the prescription error rate among intern doctors to that of their senior colleagues it seems that prescribing is a challenging task to this particular group of prescribers [3,4]. As a consequence, prescribing errors are frequently reported in hospital, and error rates ranging from 8% to 31% have been identified in studies conducted in the United Kingdom [3], Scotland [5], and Ireland [6]. It is therefore understandable that a considerable body of research has focused on this target group and there is a need for better education to prepare the medical students to meet the expectations of practice [2].

The education of medical students has been criticised for being outdated and insufficient in aligning to the realities and expectations of clinical practice [2,5,7,8,9]. Previous studies have described the complexity of the prescribing task and signposted the need for an update in medical curricula that targets both theoretical and practical domains of prescribing [3,5,9]. In a previously published review, we brought the attention to the deficits in prescribing education for intern doctors particularly in the application of theoretical knowledge to clinical practice. In this review, we also described a potential role of the pharmacist to help overcome some of the prescribing errors through interdisciplinary learning for undergraduate pharmacy and medical students to underpin the importance of all stakeholders—doctors, nurses, and pharmacists—in the care of patients [10].

In this article, we aimed to explore the opinions of newly qualified doctors (intern doctors) on the potential role of the pharmacist in prescribing education, and their views in relation to challenges experienced in prescribing and prescribing education.

## 2. Materials and Methods

A qualitative study design was chosen as this methodology enabled us to elucidate the perspectives of participants and their understanding of a particular phenomenon, namely prescribing [11]. The study was conducted in Mercy University Hospital (MUH) in Cork, Ireland. The MUH is a 350 bed acute urban hospital and due to its university teaching commitments, clinical staff are involved in the Medical Undergraduate Training Program in association with the National University of Ireland [12]. A clinical pharmacy service is provided on most of the MUH hospital wards once or twice a week, including the reconciliation of drug charts. We recruited intern doctors using a convenience sampling strategy. Every intern doctor working at MUH in the time period 1 March to 30 April 2015 was eligible however we excluded those with previous expertise or experience in prescribing, e.g., intern doctors with a pharmacy degree. Ethical approval was granted from the Clinical Research Ethics Committee of the Cork Teaching Hospitals prior to recruitment. All participants gave their informed, written consent.

Semi-structured interviews were conducted with doctors during their internship (i.e., first post qualification) year. The interview topic guide was developed based on relevant literature and followed the techniques of semi-structured interviews in the formulation of the questions and the order of these to gain a flexibility of the topic guide that allowed for the participants to share views and experiences that were important to them. The topic guide was updated on an iterative basis to allow for emerging themes to be explored in subsequent interviews. An overview of the topics covered in the interview guide is given in Table 1.

All interviews were conducted by two researchers (C.R.H. and L.J.S./E.W.) and audio-recorded. C.R.H. asked the questions and prompted the participants whenever needed to cover the topics outlined in the topic guide, but allowed for the interviewee’s answers to determine the information given to those topics [11]. Participants were also encouraged to share relevant information at the end of the interview which had not been covered already. L.J.S and E.W. were also allowed to ask questions or prompt participants during the interview. The interviews were transcribed in full after each interview prior to anonymization by C.R.H. and verified for accuracy by L.J.S. or E.W. Data collection was continued until no new themes emerged as described by Francis’ et al. [13]. Adhering to this method, we specified a priori a sample size of 10 interviews based on the homogeneity of the group of participants with respect to the interns’ level of training and experience in prescribing. Our stopping criterion was specified to three interviews, i.e., the number of additional, consecutive interviews to be conducted with no new themes emerging [13]. C.R.H. and E.W. reached agreement on the time of which data saturation was reached based on the data analysis of the interview transcripts.

With the goal of finding and defining emerging themes across the data set, thematic analysis was performed [11]. Themes were developed in an inductive way in which the interviews were coded independently by two researchers [11]. The analysis was approached in four steps: (1) C.R.H. and E.W. familiarized themselves with the data by transcribing the interviews and coded the transcripts independently. From this, C.R.H. and E.W. developed an initial list of codes independently and, through discussion, an agreed list of codes was constructed. This list was accepted by L.J.S. and C.B., (2) C.R.H. recoded the initial 10 interviews according to this list and if any adjustments or additions were needed, these were done in agreement with E.W. No major amendments were made to the initial list of codes. The last three interviews were then coded according to the updated list of codes based on the 10 initial interviews, and data saturation was reached if these were coded completely according to the list. Data saturation was reached when all authors agreed. (3) E.W. recoded four randomly selected transcripts to evaluate the trustworthiness of the coding by C.R.H. (4) C.R.H. and E.W. reviewed and categorized the codes to identify emergent themes pertinent to the research topic. These were approved by L.J.S. and C.B.

## 3. Results

A total of 13 interviews were conducted. The majority of respondents interviewed were local graduates (*n* = 11), five were females, mean age was 25 years (SD 2.14) and they had mean of nine months working experience in hospital (SD = 0.9).

### 3.1. Factors Influencing Prescribing

The influences on intern doctors’ prescribing pertained to education on prescribing, the environment, and interprofessional relationships.

### 3.2. Education on Prescribing

Three sub themes pertaining to their medical education were identified; namely curriculum content, timing, and delivery.

#### 3.2.1. Curriculum Content

Doctors felt under prepared in the practical aspects of prescribing. Suggested content was learning which focused on dosing, frequency, and duration of medical therapies; side effects; and drug interactions.

“You’re not taught enough of interactions that might be the biggest thing coming in as a doctor.” *Intern doctor (ID) 2*. “No kind of sessions about in practice prescribing” *ID 12*. “Dosing, frequency of dosing etc. is often things we have to learn on the wards.” *ID 1*.

It was also suggested that specific teaching should be provided in the areas of commonly prescribed drugs and prescribing in specific patient groups, e.g., children and the elderly.

“I don’t think we got enough training in prescribing in elderly, because I think that is a completely separate, that should be a completely separate subject, a module.” *ID 4*. “I think it’s difficult, particularly starting antibiotics, where they’re weight-based dosage. And I think we're poorly educated on that.” *ID 1*.

Pertinent legislation governing prescribing and timely introduction to the hospital drug Kardex™ and forms were also mentioned.

“It’s definitely helpful to, to go through the rules about like, you know what drugs have to go on separate prescriptions and controlled drugs, and even just bringing out see the form that it has to go on.” *ID 10*. “And I know every hospital is different with the Kardexes™, but if you’re not used to write in any Kardex™ and even like to do prescriptions properly, then it’s kind of difficult.” *ID 11*.

The doctors reported difficulties in using prescriber guidelines, such as the British National Formulary [14]. The correspondence of trade names to their generic (international non-proprietary name, INN) names was also an issue.

“We prescribed everything generically. Ehm, so I’d often Google brand names to get the generic name.” *ID 4*. “Cause I think sometimes looking up brand names in the BNF [British National Formulary] and MIMS [the Monthly Index of Medical Specialities Ireland is hard so that’s why I always Google them if I don’t know them.” *ID 12*.

#### 3.2.2. Timing

To make a better use of the early post qualification training period in hospital and general practice, it was suggested that medical students are, firstly, introduced to prescribing during pre-qualification clinical placements and, secondly, to be given more responsibility for prescribing during their education, pre-qualification.

“We get a bit of education, on the same time you don’t have too much, like, not really a student isn’t going to be prescribing, but we don’t have too much exposure to it before actually working. Now we are, I suppose, encouraged to have a look at the Kardexes™ as we’re going on placement. But apart from that it’s a little novice starting off.” *ID 7*.

The interns were aware of the legal constraints pertaining to prescribing, but felt that earlier responsibility would enhance learning. Suggested methods of introducing prescribing at an earlier stage would be allowing students to transcribe information and to have a senior doctor sign the prescription, or to go through prescriptions with recently qualified doctors during placement.

“I know you can’t properly prescribe as a medical student but you’re in a hospital for three or four years and you’re literally just following people around without ever looking at a prescription and then you go out your first day as an intern and your primary job is nearly that.” *ID 8*. “You know, as an intern you’re going to have to do so much prescribing anyway. You know. You could suggest that senior members would team you but that’s not really practical.” *ID 3*.

In addition to more training and repetition, the doctors expressed a need for receiving the training closer to the time of actually prescribing in practice. It was suggested that the training in prescribing should start early in their undergraduate education, be repeated throughout all years and continued in postgraduate training.

“They tried to teach us as much as, as they could but it’s just. I think, like that, you need more—it’s a once off starting the year and then it’s months later before you’re actually applying it.” *ID 10*. “In entering, I think you should be doing prescribing the whole time and learning about it more because you’re nervous constantly and fearing that you are going to mess up.” *ID 2*.

#### 3.2.3. Delivery

The respondents suggested that they should shadow recently qualified doctors on a regular basis during their undergraduate placement years rather than receiving only consultant-led teaching.

“We are better off shadowing an intern I think in fifth year as opposed to shadowing consultants (...) if you’re shadowing the intern you know it all better when you come out.” *ID 8*.

The engagement of near peers in the undergraduate education was believed to positively add to the clinical relevance of the education by giving the medical students an idea of what would be expected of them as newly qualified doctors. Senior house officers (SHOs) and hospital pharmacists were other suggested co-educators in prescribing.

“...if someone like an intern or an SHO (sic) would’ve come into college and shown to us a drug Kardex™ and said, look, this is what will happen, someone will ask you to do this. It’s easy to learn but nobody has ever actually shown us like, this is kind of what you will be doing on day one, and people just kind of expect you to know it when you come in to hospital.” *ID 6*. “Just be a bit more practical [education in prescribing] and probably say even from, maybe even if the hospital pharmacist came in and talked to them as well, ‘cause they’re probably better even at doing them [prescriptions].” *ID 11*.

### 3.3. Pharmacist

A subtheme pertaining to interprofessional relationships was the role of the pharmacists. The intern doctors welcomed help from pharmacists and believed that interprofessional education with pharmacy students would improve their prescribing practice and future collaboration.

“We had one session in final med that we all thought was really good. It was in conjuction with the pharmacy students and we came in and like we went through ehm, a chart (…) I suppose you’re coming at it from two different perspectives. Whereas we’d [ntern doctors] be kind of, like, ‘oh look, this is what would be commonly prescribed in here’ and what you’ve heard of. And the pharmacy students they’re coming at if from a much more kind of chemical perspective and they know about the compounds, they know how they work. And I found that we complemented each other on the day. Like, we both added to the patient’s care.” *ID 12.* “We don’t get enough interaction and training with the pharmacists that we should do. I think, there’s a lot of stuff that we should do together because of all the medical patients you’re mostly working together with the pharmacists (…) We are kind of trained to do your own thing. You don’t ask any other medical profession for help.” *ID 2*.

Pharmacists on wards and the staff in the hospital pharmacy were believed to check drug charts and prescriptions before giving drugs to the patients, and to make contact with the responsible doctor for any clarifications or corrections needed.

“I’m trying to think of times when the pharmacist would have ringed me, ‘cause that’s when it [making a prescribing error] would have been.” *ID 11*.

Pharmacists and senior colleagues were thought to be useful and available information sources when prescribing. However, some of the interns reported little interaction with pharmacists, describing occasional chance contact but hesitating to formally seek their assistance. Some reported the belief that pharmacists were an underutilised resource.

“If I saw someone [pharmacist] on the ward I would ask a question, but I don’t think I’ve ever rung, I might have ringed up once or twice ever.” *ID 10*. “I’m sure if there’s something the pharmacist would help me out with, but I haven’t gotten any phone calls back yet about anything, so.” *ID 8*.

### 3.4. Summary of Findings

**Figure 1:**
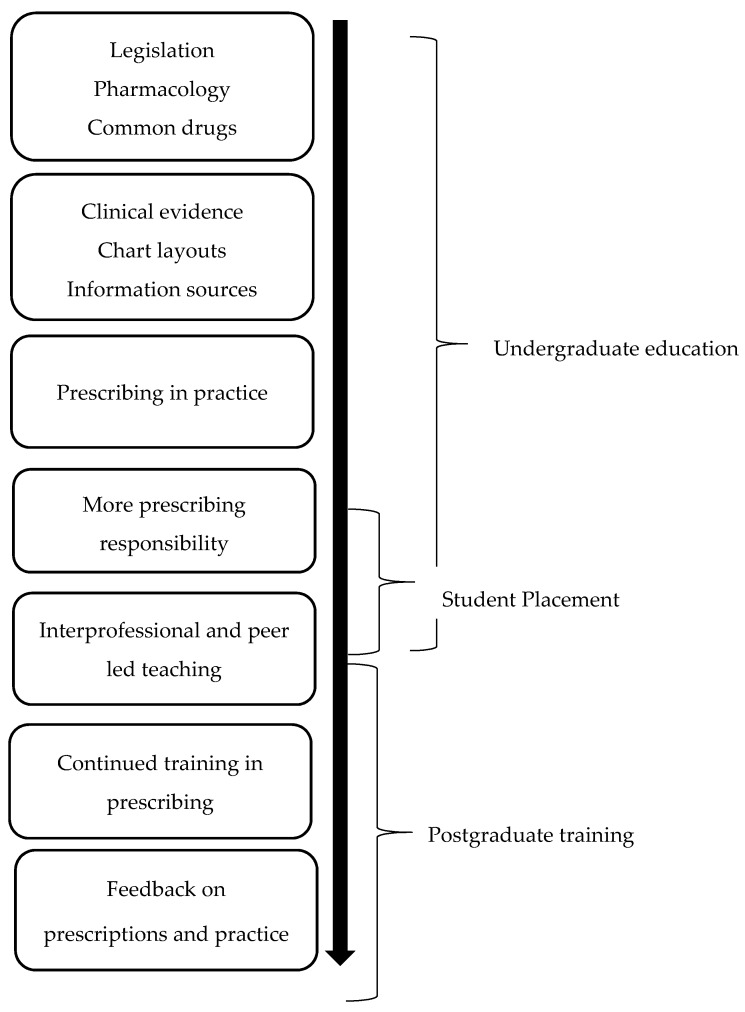
Suggested improvements to teaching in prescribing.

There is a need to adjust the delivery, timing, and content of teaching in prescribing, and to improve the integration of the pharmacist in both undergraduate and postgraduate prescribing education to raise the awareness of their potential role in safe prescribing. From the findings of this study, we suggest the improvements illustrated in Figure 1, to improve teaching in prescribing. We further want to highlight the potential role of the pharmacists in undertaking and being involved in many of these improvements, e.g., interprofessional teaching and giving feedback on prescriptions and practice to intern doctors.

## 4. Discussion

Intern doctors feel that they lack practical knowledge of prescribing, and medical education is deficient in delivering experiential exposures that enable performance of actual prescribing. This has been well established in the literature [7,8] and confirmed by our findings.

There is a limited knowledge of dosage, formulations, frequency and duration of treatment amongst this cohort [3,5,8]. Additionally, they report difficulties in applying/transferring the knowledge gained in medical school to clinical practice. Safe-prescribing skills and awareness of prescribing errors have been suggested as core components of training in prescribing in combination with supervision in the earlier years of postgraduate training [7]. The PROTECT study showed that prescribing error rates failed to improve in more experienced doctors (specialists, senior doctors, and consultants) compared to newly qualified doctors (Foundation year 1 and year 2 doctors, i.e., intern doctors in their first and second year post-graduation, respectively). The study described that only an initial lack of knowledge when prescribing can be resolved by experience, and poor prescribing by newly qualified doctors should partly be addressed by providing better feedback to the individual prescriber [5]. Hence, directing efforts to improve the training in prescribing as suggested in our study and the PROTECT study may provide a useful way of preventing prescribing errors, not only in the doctors’ first years of post-qualification but also when progressing into more senior levels. Improved knowledge may not be the single solution, and directing efforts to improve the doctors’ information-seeking skills may also be of benefit to safe prescribing. Intern doctors expressed challenges of using existing clinical guidelines (e.g., BNF) based on approved drug names. In the UK based EQUIP study the foundation year 1 doctors (i.e., intern doctors) reported that the information needed was not readily available from the BNF, and they were more likely to seek information from senior colleagues [8]. The differences in the information seeking strategies may to some extent be explained by a development in online information sources and clinical guidelines since the EQUIP study from 2009 and to our study at present. Intern doctors interviewed in our study often referred to the use of an online edition of the BNF and the use of search tools such as Google.

In addition to existing findings from the literature, our study describes new strategies to bring clinical relevance to the medical curriculum, by rethinking not only the content, as previous studies have focused on, but also the timing and the delivery of the education. Ideas to improve teaching in prescribing include: introducing the medical students to the prescribing task earlier in their undergraduate education and having peers and pharmacists take part in delivering education on prescribing. Based on our findings, we suggest working in a team environment as a postgraduate could have a positive impact on learning to prescribe safely and that such training may be valuable resource for postgraduate training that has been underutilised to date.

Some of the interns in our study welcomed the help from pharmacists, and more interaction with them was suggested to improve prescribing. From interviewing intern doctors, it seems that more frequent and better interaction with pharmacists could potentially reduce the need for a safety net to catch prescribing errors, and instead prevent rather than correct errors. Enhancing the interprofessional relationship could be beneficial to prescribing, as both professions would add to the prescribing practice, as suggested by some of the interns interviewed. The perceived role of the pharmacist as a potential collaborator in preventing errors in the process of prescribing is different to the findings of the PROTECT study, in which the pharmacist was perceived as the ‘main defence for identifying errors’ on existing prescriptions [5]. The differences in the perception of the pharmacist may be explained by the different levels of clinical pharmacy services provided in the two study settings. Local practice in the PROTECT study hospitals was prescription charts review by clinical pharmacists for prescribing errors, whereas in the hospital setting in our study, the pharmacy service provided did not officially include checking and correction of prescription errors.

We suggest that improving this interdisciplinary prescribing practice could result from a change in the hospital environment by recruiting more pharmacists to the wards and offering a pharmacy ward service more than once or twice a week. The impact of pharmacist-facilitated discharge procedures has shown to be positive, with a reduction of preventable ADEs for patients after hospitalisation and a decrease in the rate of medication-related visits to the hospital emergency departments [15]. Introducing interdisciplinary education in the medical school to pharmacy students and medical students could also improve their collaboration by an early understanding of each other’s roles in patient safety, as suggested in another study undertaken in MUH by McCague et al. (2015) [16]. A comparison of the course content in pharmacy and medicine might provide useful information. Merging the standards set by the accreditation authorities in pharmacy and medicine (the Pharmaceutical Society of Ireland, PSI; and the Irish Medical Council, IMC, respectively) could be a useful way of guiding the education in safe prescribing to both pharmacy and medicine students to improve their interdisciplinary collaboration. The PSI competencies pertaining to the safe and rational use of medicines could be applied in medicine [17]. In Ireland, the PSI and the IMC are currently working collaboratively to develop guidance on issues of joint concern to both medical practitioners and pharmacists. In a timely and relevant initiative, the two authorities have recently developed a joint guide on the Safe Prescribing and Dispensing of Controlled Drugs [18].

When interviewed about topics related to their professionalism, as with prescribing practice, it is likely to have an impact on the respondent’s answers to appear more socially acceptable [19].

The group of participants were all local graduates and all working in the same hospital. This may limit the transferability of our findings. Whilst the qualitative studies are not designed to generalise, our study has illuminated questions of relevance to intern doctors’ prescribing practices. Our findings however have demonstrated consistency with previous larger studies [2,3,4] and this may suggest that the novel findings of this study are transferable to an extent.

Prescribing safely is a skill and the successful acquisition of this skill appears to be lost at the interface of undergraduate education and postgraduate clinical practice. The newly qualified doctors themselves believe prescribing is best learned by performing the actual task and that a lack of knowledge can be resolved by practical experience of writing prescriptions. Continuing guidance is needed through a doctor’s first experiences of prescribing in clinical practice. The pharmacist may have a role in enhancing the teaching of prescribing and as a useful collaborator in improving safe prescribing practice. This study supports such collaborative engagement and that such engagement in relation to development of the undergraduate and postgraduate curriculum would be of benefit. Intervention studies are needed to assess the outcomes of changes made to the medical curriculum to further understand the specific needs of medical students and newly qualified doctors pertaining to safe prescribing.

## Figures and Tables

**Table 1:** Summary of the interview topic guide.

Topic	Description
Demographics	Gender, age, university of graduation, experience, and frequency of prescribing
Prescribing behaviour	The process and location of prescribing and associated difficulties/challenges
Information sources	Type of sources used, availability of these and ability to use them. Communication with pharmacists and peers
Education	Preparedness for the prescribing task, rating of the education
Future improvements	Improving training and teaching in prescribing, useful information sources, and other tools
